# Association of Diet Quality With Longevity and Successful Aging in Israeli Adults 65 Years or Older

**DOI:** 10.1001/jamanetworkopen.2022.14916

**Published:** 2022-06-01

**Authors:** Abigail Goshen, Uri Goldbourt, Yael Benyamini, Tal Shimony, Lital Keinan-Boker, Yariv Gerber

**Affiliations:** 1 Department of Epidemiology and Preventive Medicine, School of Public Health, Sackler Faculty of Medicine, Tel Aviv University, Tel Aviv, Israel; 2 Bob Shapell School of Social Work, Tel Aviv University, Tel Aviv, Israel; 3 Israel Center for Disease Control, Israel Ministry of Health, Tel Hashomer, Israel; 4 Lilian and Marcel Pollak Chair in Biological Anthropology, Sackler Faculty of Medicine, Tel Aviv University, Tel Aviv, Israel

## Abstract

**Importance:**

To our knowledge, the role of overall diet quality in successful aging has not been conclusively demonstrated.

**Objective:**

To prospectively examine the association between diet quality and longevity and successful aging in a population-based cohort of older adults.

**Design, Setting, and Participants:**

Participants in “Mabat Zahav” (the Israeli National Health and Nutrition Survey of Older Adults), an older adult cohort (aged ≥65 years) consisting of a random sample of 1770 individuals, were recruited from July 2005 to December 2006 (time 1 [T1]). Survivors of T1 were again contacted and asked to participate in a second interview. From May 2017 to June 2019 (time 2 [T2]), an extensive face-to-face interview and a functional assessment were conducted in each participant’s home in a subsample of 604 participants from T1, representing 72.7% of 820 surviving individuals who were able to complete interviews and assessments.

**Exposures:**

A 24-hour dietary recall, assessed at T1, was used to calculate scores from the 2015 version of the Healthy Eating Index (HEI-2015) (scores range from 0 [worst diet] to 100 [best diet]).

**Main Outcomes and Measures:**

Time to death, with follow-up lasting through June 2019, and successful aging. The latter, based on T2 assessment, was defined as (objectively measured) preserved physical and cognitive function and (subjective) mental well-being and favorable self-rated health. Inverse probability weighting was used in the analysis to minimize attrition bias.

**Results:**

At T1, the study included 1770 participants (mean [SD] age, 74.6 [6.2] years; 943 women [53%]). On average, participants with higher HEI-2015 scores had healthier lifestyles and higher socioeconomic status at T1. During a median follow-up duration of 12.6 years (IQR, 7.6-13.2 years), 893 deaths occurred. Among the 596 T2 participants analyzed (mean [SD] age, 84.1 [4.4] years; 334 [56%] women), 242 (40%) met successful aging criteria. After adjustment for sociodemographic and lifestyle risk factors, a higher HEI-2015 score was inversely associated with mortality (hazard ratios, 0.85; 95% CI, 0.72-0.99 in the upper tertile and 0.83; 95% CI, 0.71-0.98 in the middle tertile vs the lower tertile; *P* = .04 for trend) and was positively associated with successful aging (odds ratios, 1.73; 95% CI, 1.10-2.72 in the upper tertile and 1.30; 95% CI, 0.83-2.03 in the middle tertile vs the lower tertile; *P* = .03 for trend).

**Conclusions and Relevance:**

In this cohort study of older adults in Israel, improved diet quality was associated with increased longevity and successful aging in a dose-dependent manner. These data contribute to the body of literature that suggests diet quality is associated with aging in the older age group.

## Key Points

**Question:**

What is the association between diet quality and longevity and successful aging among adults aged 80 years or older?

**Findings:**

In this cohort study of 1770 Israeli participants followed up from older adulthood, a higher diet quality score was inversely associated with mortality and positively associated with successful aging, defined as preserved physical and cognitive function and robust mental health, in a dose-dependent manner.

**Meaning:**

The findings suggest an association between diet quality and longevity and successful aging and also suggest a dose-dependent association between diet quality and both outcomes.

## Teaser

This cohort study examines the association between diet quality and longevity and successful aging in a population of Israeli adults 65 years or older.

## Introduction

Population aging is accelerating rapidly, presenting extraordinary challenges to health care systems.^1^ Eurostat^2^ forecasts that, by 2050, the proportion of the European Union population older than 65 years will increase from 16.0% in 2010 to 27.8% and the population older than 80 years, from 4.1% to 10.1%. In the United States alone, the proportion of persons 85 years or older is expected to increase from less than 2% in 2010 to more than 4% by 2050, constituting more than 20% of persons 65 years or older.^3^ Thus, the health of the fast-growing segment of the oldest-old age group (aged ≥80 years), with members who are more susceptible to disease and disability, is becoming an important public health and policy issue.^4^

Successful aging is generally described as a complex process of adaptation to changes throughout the life span with preserved health and physical, social, and mental well-being.^5^ However, this broad theoretical description should be considered in terms of a measurable outcome that can be empirically validated. In a systematic review of successful aging definitions, Cosco et al^6^ revealed 105 definitions for assessment of successful aging. A wide range (0.4%-95%) of successful aging prevalence was found by Depp and Jeste^7^ in a review of quantitative studies among community-dwelling older adults. Nevertheless, there is an increasing consensus that measurement of a successful aging outcome should include evaluation of the capacity to function well in the cognitive, physical, and mental domains, incorporating biomedical and individual perspectives in a multidimensional approach.^6^

Studies assessing determinants of longevity and successful aging have found associations with healthy lifestyle and nutrition.^8,9^ Nutrition may facilitate physical and cognitive function and ultimately promote successful aging.^10^ Measures of dietary patterns and overall diet quality indices are becoming increasingly common to assess the complex exposure dietary intake represents.^11^ The Healthy Eating Index (HEI) is such an index,^12^ developed by the US Department of Agriculture as a measure of diet quality and updated every 5 years to correspond to changes in dietary guidelines.^13^ The scoring method in the HEI’s latest version (HEI-2015) is density based (ie, on amounts per 1000 kcal) and allows both protective dietary patterns and unfavorable intakes to be identified. The HEI-2015 has also been previously used to describe diet quality in worldwide populations and subpopulations.^12,14^ Previous studies in older adults showed that a higher HEI score was associated with better survival^15^ and successful aging as defined by a lack of chronic diseases, no major functional limitations, and good mental health.^16^ However, of the studies reported to date, only a few have explicitly examined the concept of longevity and successful aging among the oldest old (aged ≥80 years). This is important because the latter group is more heterogeneous in function and might be differentially affected by unique risk factors and health-promoting factors.^17^

In the present study, a theoretically driven model was used to assess the association of diet quality with longevity and successful aging. The model included objectively measured domains of physical and cognitive function in addition to subjective assessments of mental well-being and general health. Long-term associations of overall diet quality were examined with longevity and successful aging among the oldest-old individuals in Israel.

## Methods

### Study Population

**Figure 1.**
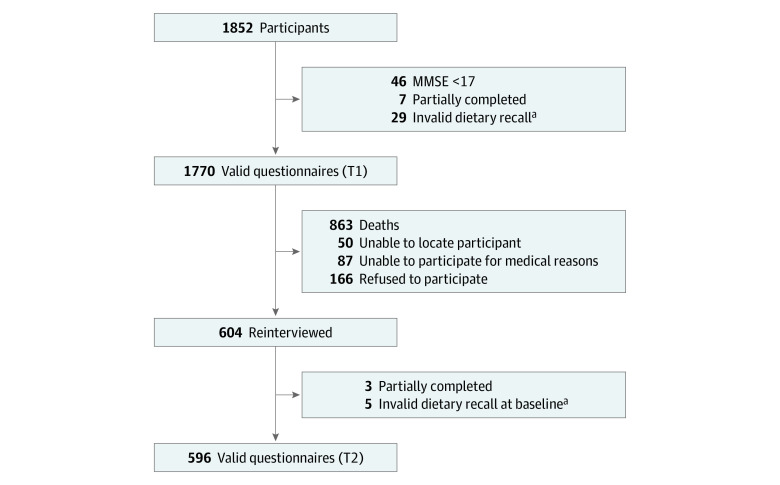
Flowchart of the Study Sample MMSE indicates Mini-Mental Status Examination; T1, time 1 (July 2005 to December 2006); and T2, time 2 (May 2017 to June 2019). ^a^Invalid dietary recall was defined as a total energy intake of less than 400 kcal/d or greater than 5000 kcal/d.

The Israeli Longitudinal Study on Aging is a population-based cohort study investigating lifestyle habits and psychosocial and general health in the context of healthy aging and adverse clinical outcomes among community-dwelling older adults in Israel. The study methods and procedures have been described elsewhere.^18^ Briefly, we studied a group of older adults who took part in “Mabat Zahav” (the first Israeli National Health and Nutrition Survey of Older Adults),^19^ which was conducted from July 2005 to December 2006 (time 1 [T1]). Survivors of T1 were contacted by telephone; those who agreed to participate and were physically capable were scheduled for a face-to-face interview at their home. The original survey population constituted a random sample of Israeli citizens 65 years or older and included 1852 participants. Exclusion criteria were cognitive impairment as measured by a Mini-Mental State Examination (MMSE)^20^ score less than 17, long-term hospitalization, and severe dementia. Among initial participants, 1770 were included in the present analysis. During the period from May 2017 to June 2019 (time 2 [T2]), an extensive face-to-face interview and a functional assessment (eFigure in the Supplement) were conducted among 604 past participants, representing 73.7% of 820 medically capable surviving individuals (survivors who were able to complete interviews and assessments). Of the 604 T2 participants, 596 (72.7% of the medically capable survivors) were included in the successful aging analysis (Figure 1). Ethical approval for the study was obtained from the Helsinki Committee of Chaim Sheba Medical Center at Tel Hashomer, and each participant signed an informed consent form at both T1 and T2. This study is reported according to the Strengthening the Reporting of Observational Studies in Epidemiology (STROBE) reporting guideline.

### Nutritional Assessment

A multiple-pass, 24-hour dietary recall was used to calculate HEI-2015 scores at T1.^12^ The HEI-2015 includes 13 dietary components^12^: 9 adequacy components (those recommended for inclusion in a healthy diet), including total fruits, whole fruits, total vegetables, greens and beans, whole grains, dairy, total protein foods, seafood and plant proteins, and fatty acids, and 4 moderation components (to be consumed sparingly), including refined grains, sodium, added sugars, and saturated fats. For each component, the respondents receive a minimum score of 0 and a maximum score of 5 or 10 (for perfect adherence). Thus, the overall index score ranges from 0 (worst diet) to 100 (best diet).

### Successful Aging Assessment

We adopted a multidimensional approach in which successful aging was defined as satisfying all of the following criteria: (1) preserved physical function, (2) preserved cognitive function, (3) favorable self-rated health, and (4) mental well-being.

Preserved physical function was assessed by the absence of frailty according to the frailty phenotype model.^21^ This model comprises 5 physical indicators, including low physical activity (lowest 20% score in the sample according to Physical Activity Scale for the Elderly Questionnaire^22^ for each sex), weak grip strength (lowest 20% in the sample adjusted for sex and body mass index [BMI]), slow walking speed (slowest 20% in the sample, based on time of a 5-m walk adjusted for sex and standing height), self-reported exhaustion, and unintentional weight loss (>4.5 kg or >5% of body weight in the previous year). Frailty was defined by the presence of more than 2 indicators.

Preserved cognitive function was assessed by a lack of substantial decline (≤1 SD, equivalent to a 4-point reduction) as defined by change in MMSE^20^ scores between the 2 interviews.

Favorable self-rated health was assessed by a single question asked at T2^23^: “How would you rate your health status?” A 4-point scale was used. A rating of excellent or good (compared with fair or poor) was considered favorable.

Mental well-being was assessed as the absence of depression. Risk of depression was evaluated via a 5-item short form of the Yesavage Geriatric Depression Scale.^24^ A score higher than 2 indicated possible depression.^24^

### Mortality Assessment

Death during follow-up was updated through June 2019; original participants were linked to the nationwide database of causes of death via their national identification numbers. The Israeli Ministry of Health manages mortality information.

### Additional Covariates

Educational level was divided into 2 categories: 0 to 12 years and more than 12 years of schooling. Physical function was assessed by the Katz Index of Independence in Activities of Daily Living (Katz ADL) scale.^25^ The maximum score is 15, with a score of 6 or lower indicating no functional limitations. Physical activity engagement was based on a general question of whether the participant engaged in regular physical exercise long enough to work up a sweat at least once a week (yes or no). Smoking status was classified as current, past, or none.

### Statistical Analysis

Baseline characteristics of study participants by tertiles of HEI-2015 score were compared by using the χ^2^ test for categorical variables and analysis of variance for continuous variables. Survival analysis was performed by using Cox proportional hazards models^26^ to estimate the association (hazard ratios [HRs] and 95% CIs) between HEI-2015 and mortality. The proportional hazards assumption was tested using Schoenfeld residuals and was met in all models. Multivariable logistic regression models were used to examine the association (odds ratios [ORs] and 95% CIs) between baseline HEI-2015 scores and successful aging at T2. The HEI-2015 score was modeled either categorically (tertiles) or as a continuous variable. The covariates in the regression models were selected a priori as potential confounders and included sex, age, educational level, physical activity, and smoking status. We attempted to avoid overadjustment for intermediate variables on the potential causal path from HEI-2015 to outcome.^27,28,29^

Of the initial participants, many were unable to attend the second interview. We used inverse probability weighting^30^ to minimize the repercussions of attrition-related selection bias for the estimated association between HEI-2015 and successful aging. The weights were calculated using a multinomial logistic regression model to assess the probability of original participants of attending T2. For higher specificity, we distinguished between attrition due to mortality and attrition due to other causes (eg, refusal to participate, unsuccessful contact).^31^ Each observation was then weighted by the reciprocal of the estimated probability of participating at T2. Because of the instability that extreme weights can induce, stabilized weights were used.^32^ Missing values for components of the successful aging outcome and model covariates were imputed using multiple imputation methods.^33^ Accordingly, 5 data sets were created, with missing values replaced by imputed values based on models incorporating demographic, socioeconomic, psychosocial, and clinical variables as covariates. The results of these data sets were then combined using the Rubin rules.^33^ Owing to a high percentage of missing values in the frailty phenotype index (14%), we also assessed an alternative successful aging model that included preserved physical function through the Katz ADL scale rather than the frailty phenotype model.

Analyses were performed using SAS, version 9.4 (SAS Institute Inc); SPSS, version 27 (IBM); and R, version 3.4.4 (R Foundation for Statistical Computing). A 2-sided *P* ≤ .05 was considered statistically significant.

## Results

Among the 1770 T1 participants, the mean (SD) age was 74.6 (6.2) years, 943 (53%) were women, and 827 (47%) were men. The HEI-2015 scores at baseline ranged from 13 to 94 (mean [SD], 60.0 [13.0]). Baseline characteristics of participants by HEI-2015 tertiles are given in Table 1. On average, participants with higher HEI-2015 scores were more educated and had higher household income, better self-rated health, and fewer functional limitations. They were also more physically active and had higher MMSE scores than participants with lower HEI-2015 scores.

**Table 1.** Baseline Characteristics of Study Participants by Tertiles of HEI-2015 Score at T1

Characteristic	No. (%)				*P* value
	Total (N = 1770)	Tertiles of HEI-2015 score at study entry			
		Lower (n = 590)	Middle (n = 590)	Upper (n = 590)	
HEI-2015 score, mean (SD)	60 (13.0)	45.4 (6.8)	59.7 (3.3)	74.0 (6.2)	<.001
Women	943 (53)	308 (52)	305 (52)	330 (56)	.28
Men	827 (47)	282 (48)	285 (48)	260 (44)	
Age, mean (SD), y	74.6 (6.2)	74.6 (6.4)	75.3 (6.4)	74.0 (5.8)	.002
Educational level, >12 y	610 (35)	164 (28)	201 (34)	245 (42)	<.001
Household income category^a^					
Low	759 (43)	303 (51)	241 (41)	215 (36)	<.001
Intermediate	421 (24)	110 (19)	143 (24)	168 (29)	
High	590 (33)	177 (30)	206 (35)	207 (35)	
Married	1127 (64)	358 (61)	381 (65)	388 (66)	.16
Self-rated health good or very good	979 (55)	275 (47)	325 (54)	379 (64)	<.001
No functional limitation^b^	1473 (83)	474 (80)	481 (82)	518 (88)	.008
Regular physical activity	553 (31)	127 (22)	186 (32)	240 (41)	<.001
BMI, mean (SD)	29.0 (4.8)	29.6 (5.2)	29.1 (4.8)	29.1 (4.5)	.15
MMSE score, mean (SD)	27.1 (3.5)	26.4 (5.2)	27.0 (3.5)	27.7 (2.9)	<.001
Current smoking status	190 (11)	87 (15)	68 (12)	35 (6)	<.001
No. of chronic diseases, median (IQR)	3.0 (1.0-4.0)	3.0 (1.0-4.0)	2.0 (1.0-4.0)	2.0 (1.0-3.3)	.06

Abbreviations: BMI, body mass index (calculated as weight in kilograms divided by height in meters squared); HEI-2015, 2015 version of the Healthy Eating Index; MMSE, Mini-Mental State Examination; T1, time 1 (July 2005 to December 2006).

^a^ Household income categories: low, less than 5254 new Israeli shekels (NIS); intermediate, 5255 to 10 459 NIS; and high, more than 10 460 NIS.

^b^ According to a Katz Index of Independence in Activities of Daily Living score of 6 or less.

**Figure 2.**
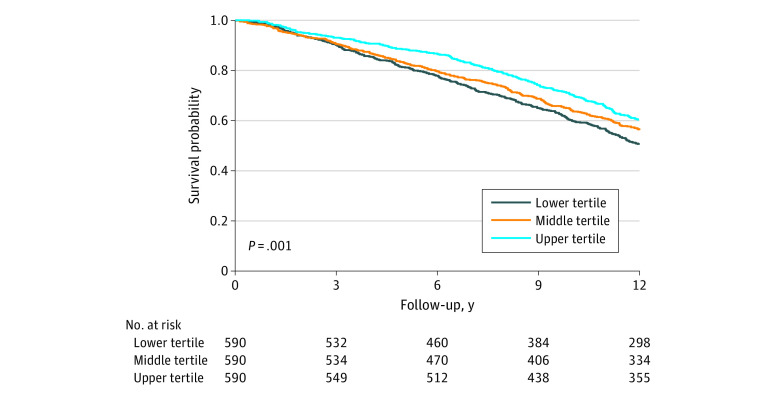
Kaplan-Meier Survival Curves During Follow-up Stratified by Healthy Eating Index 2015 Tertile

During a median follow-up of 12.6 years (IQR, 7.6-13.2 years), 893 deaths occurred. HEI-2015 scores were inversely associated with mortality (Figure 2). When adjusted for age and sex, the HRs for mortality were 0.76 (95% CI, 0.64-0.89) in the upper tertile and 0.80 (95% CI, 0.68-0.93) in the middle tertile compared with the lower tertile (*P* < .001 for trend). Further adjustment for educational level, physical activity, and smoking status did not substantially change the association (HRs, 0.85; 95% CI, 0.72-0.99 in the upper tertile and 0.83; 95% CI, 0.71-0.98 in the middle tertile; *P* = .04 for trend). On a continuous scale, each 10-point improvement in HEI-2015 score was associated with a multivariable-adjusted HR of 0.95 (95% CI, 0.90-1.00).

In all, 1174 (66%) of T1 participants did not attend T2 (Figure 1). A comparison of baseline characteristics of T2 participants and nonparticipants is presented in Table 2. The mean (SD) age of participants at T2 was 84.1 (4.4) years, 334 (56%) were women, and 262 (44%) were men. Overall, 242 participants (40%) in T2 met all criteria for successful aging (eTable 1 in the Supplement). On average, T2 participants who met the criteria for successful aging had higher levels of education, higher household income, and higher HEI-2015 scores and were more physically active than participants who did not meet the criteria and nonparticipants at T2 (Table 2). After multivariable adjustment, HEI-2015 scores were associated with increased ORs for successful aging: 1.73 (95% CI, 1.10-2.72) in the upper tertile and 1.30 (95% CI, 0.83-2.03) in the middle tertile compared with the lower tertile (*P* = .03 for trend) (Table 3). When individual successful aging components were assessed, higher HEI-2015 scores were associated with grip strength, cognitive function, self-rated health, and mental health (eTable 2 in the Supplement). Modeled as a continuous variable, each 10-point increase in HEI-2015 score was associated with an age- and sex-adjusted OR of 1.17 (95% CI, 1.02-1.35) for successful aging and 1.13 (95% CI, 0.98-1.31) in the fully adjusted model.

**Table 2.** Comparison of Baseline Characteristics of T2 Participants and Nonparticipants

Baseline variable	No. (%)				*P* value
	Died (n = 863)	Nonsuccessful aging (n = 354)^a^	Successful aging (n = 242)^b^	Missing data (n = 311)^c^	
Age, mean (SD), y	77.3 (6.6)	72.6 (4.9)	71.2 (4.2)	72.6 (4.9)	<.001
Women	411 (48)	196 (55)	138 (57)	198 (64)	<.001
Men	452 (52)	158 (45)	104 (43)	113 (36)	
Educational level, mean (SD), y	10.2 (5.1)	9.8 (5.1)	12.8 (4.6)	11.1 (5.5)	<.001
Household income category^d^					
Low	396 (46)	147 (42)	61 (34)	147 (47)	<.001
Intermediate	191 (22)	80 (23)	76 (31)	74 (24)	
High	276 (32)	127 (36)	105 (43)	90 (29)	
Married	504 (58)	256 (72)	183 (76)	184 (59)	<.001
Comorbidities, No.					
0-1	130 (15)	106 (30)	90 (37)	55 (18)	<.001
2-3	300 (35)	168 (48)	125 (52)	146 (47)	
≥4	433 (50)	80 (23)	27 (11)	110 (35)	
BMI, mean (SD)	29.1 (5.2)	29.4 (4.5)	28.9 (4.2)	29.3 (4.5)	.03
MMSE score, mean (SD)	26.4 (3.9)	27.1 (3.5)	28.6 (1.7)	27.4 (3.0)	<.001
Regular physical activity	217 (25)	111 (31)	114 (47)	106 (34)	<.001
No functional limitation^e^	631 (73)	327 (92)	234 (97)	281 (90)	<.001
HEI-2015 score, mean (SD)	58.8 (12.9)	60.4 (12.6)	63.4 (12.9)	59.3 (12.5)	<.001

Abbreviations: BMI, body mass index (calculated as weight in kilograms divided by height in meters squared); HEI-2015, 2015 version of the Healthy Eating Index; MMSE, Mini-Mental State Examination; T2, time 2 (May 2017 to June 2019).

^a^ Did not meet criteria for successful aging.

^b^ Met criteria for successful aging.

^c^ Participants who declined to participate at T2, could not be located, or could not participate owing to exclusion criteria.

^d^ Household income categories: low, less than 5254 new Israeli shekels (NIS); intermediate, 5255 to 10 459 NIS; and high, more than 10 460 NIS.

^e^ According to a Katz Index of Independence in Activities of Daily Living score of 6 or lower.

**Table 3.** Weighted Odds Ratios (95% CIs) of Successful Aging According to Tertiles of HEI-2015 Scores Among 596 Study Participants at T2^a^

Adjustment	Odds ratio (95% CI) by HEI-2015 score tertile			*P* value for trend
	1st	2nd	3rd	
Model 1^b^	1	1.35 (0.88-2.07)	1.93 (1.25-2.98)	.003
Model 2^c^	1	1.30 (0.83-2.03)	1.73 (1.10-2.72)	.03

Abbreviations: HEI-2015, 2015 version of the Healthy Eating Index; T2, time 2 (May 2017 to June 2019).

^a^ Inverse probability weights were applied to all analyses.

^b^ Adjusted for age and sex.

^c^ Further adjusted for educational level, physical activity, and smoking status.

We conducted several sensitivity analyses to test the robustness of our findings. First, because we applied the inverse probability weighting method in the main analysis, an unweighted analysis was also performed and yielded similar results (eTable 3 in the Supplement). Second, a subset analysis was performed among participants with no functional or cognitive limitations at baseline (eTable 4 in the Supplement). Although some attenuation was noted in the association between HEI-2015 scores and successful aging, this analysis suggests that reverse causality may not be the main explanation for the observed association. Third, we tested an alternative successful aging model in which preserved physical function was evaluated through the Katz ADL scale (score ≤6) instead of lack of frailty. In this analysis, 390 participants (65%) met the criteria for successful aging, and the ORs for successful aging were similar to those in the main analysis (eTable 5 in the Supplement). Fourth, further adjustment for household income, BMI, and comorbidities did not substantially alter the results (eTable 6 in the Supplement), nor did the application of different definitions to the covariates adjusted for in the main analysis.

## Discussion

In this cohort study of Israeli older adults, higher diet quality was associated with longevity and successful aging in the subsequent 12 years. Furthermore, a dose-dependent association with diet quality was suggested for both outcomes. These data contribute to an increasingly large body of literature suggesting that diet quality is associated with aging in the oldest-old age group.

Our results suggest that the association between diet quality and mortality, as previously demonstrated among younger age groups using different diet quality measures (eg, Alternate Mediterranean Diet, Dietary Approaches to Stop Hypertension),^34^ is maintained in the oldest-old age group. Our findings also support the recommendations of the 2015 Dietary Guidelines Advisory Committee that it is not necessary to conform to a single diet plan to achieve healthy eating patterns.^35^

One of the most studied dietary patterns is the Alternate Mediterranean Diet, which consists mainly of a high intake of fish, vegetables, legumes, fruits, cereals, and unsaturated fatty acids and is typical of some Mediterranean regions.^36^ Several cohort studies showed that greater adherence to the Alternate Mediterranean Diet was associated with survival and successful aging in older adults.^37^ However, these associations were not as clear among studies conducted in non-Mediterranean countries.^38^ Therefore, questions have been raised about the translation of the Alternate Mediterranean Diet and whether its related outcomes are generalizable to other countries and cultures.^39^ In the present study, diet quality was assessed by the level of adherence to national dietary guidelines. Hence, our findings might be more applicable to older adults in Western countries because there is a high level of consistency across countries on several dietary recommendations.^40^

In the present study, approximately 40% of T2 participants met the criteria for successful aging. Participants who met the criteria for successful aging had higher socioeconomic status and healthier lifestyle habits than those who did not, which is in agreement with previous cohort studies.^16,41^ A systematic review by Cosco et al^6^ suggested a mean successful aging prevalence of 33% (95% CI, 24%-43%) among different older adult populations. Cho et al^42^ conducted a study among the oldest-old age group in the United States and defined successful aging according to subjective health status and perceived happiness. They identified 62% of study participants as meeting the criteria for successful aging. On the other hand, von Faber et al,^43^ in Leiden’s 85-Plus Study conducted in the Netherlands, defined successful aging as the absence of disability, depressive symptoms, and cognitive impairment and found a successful aging rate between 9% and 16%. Thus, it is evident that the absence of disease or disability is the most demanding criterion for measurement of successful aging. Disease and some functional deterioration are almost inevitable in very old age. In the present study, only 9% of the participants at T2 had reported the absence of any chronic disease.

A systematic review of dietary patterns and different domains of successful aging supported the association between dietary intake, life expectancy, and physical and mental function in older adults.^44^ However, the review also revealed that the number of studies investigating the association between overall diet quality and multidimensional successful aging is small, suggesting the need for additional longitudinal studies, particularly among the oldest-old age group. The present study thus addresses this need, and the results suggest that a higher diet quality score is associated with increased odds of successful aging as assessed by a multidimensional approach. The multidimensional approach included domains previously recognized as crucial for defining successful aging, such as frailty,^45^ intra-individual changes in cognitive function,^46^ self-rated health,^47^ and mental well-being.^48^

Several mechanisms have been proposed for the observed associations. Previously, an inverse association between HEI-2015 score and plasma biomarkers of chronic inflammation was reported.^49^ In addition, healthy dietary patterns usually include increased consumption of vegetables, fruits, legumes, nuts, and seeds, which provide a range of nutrients including B vitamins, ω-3 fatty acids, fiber, flavonoids, and antioxidants, thus inducing antioxidant activity and inhibition of inflammation and angiogenesis.^34^ Other mechanisms should be the focus of future investigations.

### Strengths and Limitations

Strengths of this study include its prospective design with an extended follow-up duration and a large and rich database with high follow-up rates in a unique, nationally representative population segment of oldest-old adults. In addition, the use of the latest edition of the HEI (HEI-2015) and the comprehensive definition of successful aging are novel. Nevertheless, this study has certain limitations. First, the HEI-2015 score was based on a single 24-hour dietary recall, an approach that may be insufficient to reflect usual or rarely eaten foods over a long period and may result in a substantial regression-attenuation bias. The 24-hour dietary recall tool has been widely used to assess dietary intake in population studies,^50^ and the multiple-pass 24-hour dietary recall technique limits the extent of underreporting that occurs with a single self-reported food intake.^51^ In addition, the evaluation of the HEI-2015 score is suitable for a single 24-hour dietary recall.^52^ Nevertheless, the HEI-2015 is not age specific, and its score is expressed as a function of total energy intake, which might be overestimated or underestimated. Second, although we were able to adjust for several important potential confounders, some residual confounding is likely. Third, T2 participants differed in several baseline characteristics from nonparticipants, primarily as a result of attrition. We attempted to address this methodological challenge by applying inverse probability weighting. Some data were missing, and a multiple-imputation method was applied to overcome this. We also performed a series of sensitivity analyses that supported the results of the main analysis, including a subset analysis of the association between HEI-2015 and successful aging among more robust participants at baseline to assess the extent of a possible reverse association.

## Conclusions

The results of this cohort study support an association between a healthier diet, higher physical and cognitive function, better mental health, and reduced mortality in the oldest-old age group. Although a complete understanding of the association between nutrition and health outcomes has remained elusive, the available information highlights the need for public health intervention with a “never too late” attitude. Raising awareness of the future benefits of healthy food choices, in addition to providing healthy alternatives and dietary education, could lead to successful aging outcomes in the older adult population.

## Notes

Supplement. **eTable 1.** Prevalence of Each SA Domain Among T2 Participants **eTable 2.** Mean HEI-2015 Score at Baseline According to Preserved/Not Preserved Status of Individual SA Components at Follow-up **eTable 3.** Unweighted Odds Ratios (95% Confidence Intervals) of SA Across HEI-2015 Tertiles Among T2 Participants **eTable 4.** Unweighted Odds Ratios (95% Confidence Intervals) of SA Across HEI-2015 Tertiles Among T2 Participants With No Functional or Cognitive Limitations at Baseline **eTable 5.** Weighted Odds Ratios (95% Confidence Intervals) of Alternative SA Across HEI-2015 Tertiles Among T2 Participants **eTable 6.** Weighted Odds Ratios (95% Confidence Intervals) of SA Across HEI-2015 Tertiles Among T2 Participants Applying a Different Adjustment Approach **eFigure.** General Study Design
