# Supplementary material for: Association of Diet Quality With Longevity and Successful Aging in Israeli Adults 65 Years or Older
Source: JAMA Netw Open. 2022 Jun 1;5(6):e2214916. doi: 10.1001/jamanetworkopen.2022.14916 (PMC9161011; doi:10.1001/jamanetworkopen.2022.14916)
Supplement: Supplement. — eTable 1. Prevalence of Each SA Domain Among T2 Participants eTable 2. Mean HEI-2015 Score at Baseline According to Preserved/Not Preserved Status of Individual SA Components at Follow-up eTable 3. Unweighted Odds Ratios (95% Confidence Intervals) of SA Across HEI-2015 Tertiles Among T2 Participants eTable 4. Unweighted Odds Ratios (95% Confidence Intervals) of SA Across HEI-2015 Tertiles Among T2 Participants With No Functional or Cognitive Limitations at Baseline eTable 5. Weighted Odds Ratios (95% Confidence Intervals) of Alternative SA Across HEI-2015 Tertiles Among T2 Participants eTable 6. Weighted Odds Ratios (95% Confidence Intervals) of SA Across HEI-2015 Tertiles Among T2 Participants Applying a Different Adjustment Approach eFigure. General Study Design [file jamanetwopen-e2214916-s001.pdf]

Supplementary Online Content

Goshen A, Golbourt U, Benyamini Y, Shimony T, Keinan-Boker L, Gerber Y. Association of diet quality with longevity and successful aging in Israeli adults 65 years or older. *JAMA Netw Open*. 2022;5(6):e2214916. doi:10.1001/jamanetworkopen.2022.14916

eTable 1. Prevalence of Each SA Domain Among T2 Participants

eTable 2. Mean HEI-2015 Score at Baseline According to Preserved/Not Preserved Status of Individual SA Components at Follow-up

eTable 3. Unweighted Odds Ratios (95% Confidence Intervals) of SA Across HEI-2015 Tertiles Among T2 Participants

eTable 4. Unweighted Odds Ratios (95% Confidence Intervals) of SA Across HEI-2015 Tertiles Among T2 Participants With No Functional or Cognitive Limitations at Baseline

eTable 5. Weighted Odds Ratios (95% Confidence Intervals) of Alternative SA Across HEI-2015 Tertiles Among T2 Participants

eTable 6. Weighted Odds Ratios (95% Confidence Intervals) of SA Across HEI-2015 Tertiles Among T2 Participants Applying a Different Adjustment Approach

eFigure. General Study Design

This supplementary material has been provided by the authors to give readers additional information about their work.

© 2022 Goshen A et al. *JAMA Network Open*.

eTable 1. Prevalence of each SA domain among T2 participants (n=596).

SA domain	n (%)	Missing data, n (%)
Preserved physical function	516 (87)	86 (14)*
Preserved cognitive function	479 (80)	0 (0)
Good self-reported health	390 (65)	7 (1)
Good mental health	486 (82)	15 (2)**

Notes: MMSE= mini-mental state examination; SA= successful aging; n= number. Presented are prevalence of each successful aging domain among follow-up participants. *At least one missing indicators of the frailty phenotype. **Incomplete questionnaire of the Yesavage geriatric depression form.

© 2022 Goshen A et al. *JAMA Network Open*.

eTable 2. Mean HEI-2015 score at baseline according to preserved/not preserved status of individual SA components at follow-up.

**SA component**	**Mean HEI score (SD)**		
	Preserved	Not preserved	P-value
Physical activity level	61.73 (12.75)	60.95 (13.13)	.55
Grip strength	62.25 (12.65)	58.85 (13.21)	.005
Walking speed	61.51 (12.74)	61.79 (13.08)	.81
Self-reported energy	61.15 (12.86)	61.75 (12.81)	.60
Weight	61.20 (12.87)	62.42 (12.69)	.29
Cognitive function	62.08 (12.85)	59.68 (12.51)	.06
Self-rated health	62.27 (12.93)	60.15 (12.50)	.05
Mental health	62.28 (12.96)	58.91 (12.20)	.01

Notes: SA= successful aging; HEI= healthy eating index. Presented mean HEI-2015 score at baseline according to preserved\not preserved status of individual SA components at follow-up. Physical activity: score based on a Physical Activity Scale for the Elderly (PASE) questionnaire. Grip strength: an average of 2 ,measurement in Kg. Walking speed: time (sec) of a 5-meter walk. Self-reported energy: How much of the time during the past 4 weeks did you have a lot of energy? (All of the time-1, Most of the time-2, A good bit of the time-3, Some of the time-4, A little of the time-5, None of the time-6). Weight: Unintentionally weight loss of more than one kilogram in the past 6 months? (yes=1, no=0). Cognitive function: defined by change in MMSE scores between the two interviews. Self-rated health: "How would you rate your health status?" on a 4-point scale. Mental health: a 5-item short form of the Yesavage Geriatric Depression Scale (GDS).

© 2022 Goshen A et al. *JAMA Network Open*.

eTable 3. Unweighted odds ratios (95% confidence intervals) of SA across HEI-2015 tertiles among T2 participants (n=596).

Adjustment	***Healthy eating index score tertile***			
	1st	***2nd***	***3rd***	***P for trend***
Model 1	1	1.34 (0.88-2.06)	1.96 (1.29-2.99)	.002
Model 2	1	1.28 (0.83-1.98)	1.72 (1.10-2.68)	.02

Notes: SA= successful aging; HEI= healthy eating index. Presented unweighted odds ratios of SA across HEI-2015 tertiles among T2 participants (n=596).
Model 1: Adjusted for age and sex. Model 2: Further adjusted for education, physical activity, and smoking.

eTable 4. Unweighted odds ratios (95% confidence intervals) of SA across HEI-2015 tertiles among T2 participants with no functional or cognitive limitations at baseline (n=452).

Adjustment	***Healthy eating index score tertile***			
	1st	***2nd***	***3rd***	***P for trend***
Model 1	1	1.10 (0.67-1.81)	1.75 (1.08-2.84)	.02
Model 2	1	1.05 (0.63-1.76)	1.54 (0.93-2.57)	.09

Notes: SA= successful aging; HEI= healthy eating index.*According to cut point of ADL score ≤6 and MMSE score ≥27. Presented unweighted odds ratios of SA according to tertiles of the HEI among T2 participants with no functional or cognitive limitations at baseline (n=452).
Model 1: Adjusted for age and sex. Model 2: Further adjusted for education, physical activity, and smoking.

© 2022 Goshen A et al. *JAMA Network Open*.

eTable 5. Weighted odds ratios (95% confidence intervals) of alternative SA across HEI-2015 tertiles among T2 participants.

Adjustment	*Healthy eating index score tertile*			
	1st	*2nd*	*3rd*	*P for trend*
Model 1	1	1.43 (0.91-2.24)	2.03 (1.30-3.17)	.002
Model 2	1	1.34 (0.84-2.12)	1.74 (1.09-2.77)	.02

Notes: SA= successful aging; HEI= healthy eating index. Presented odds ratios of Alternative successful aging (n=390) according to tertiles of the HEI scores.
Model 1:Adjusted for age and sex. Model 2: Further adjusted for education, physical activity, and smoking.

eTable 6. Weighted odds ratios (95% confidence intervals) of SA across HEI-2015 tertiles among T2 participants applying a different adjustment approach (n=596).

Adjustment	*Healthy eating index score tertile*			
	1st	*2nd*	*3rd*	*P for trend*
Model 1	1	1.35 (0.88-2.07)	1.93 (1.25-2.98)	.003
Model 2	1	1.21 (0.77-1.90)	1.60 (1.01-2.52)	.03

Notes: SA= successful aging; HEI= healthy eating index. Presented odds ratios of successful aging according to tertiles of the HEI scores.
Model 1: Adjusted for age and sex. Model 2: Further adjusted for education, number of chronic diseases, physical activity, and BMI.
Number of comorbid conditions were self-reported at study entry, and included the following: cardiovascular disease, cataract, chronic renal failure, cancer, Parkinson, lung disease, diabetes mellitus, osteoporosis, hyperlipidemia, and hypertension.
Measured height and weight were used to calculate BMI (kg/m^2).

© 2022 Goshen A et al. *JAMA Network Open*.

eFigure. General study design

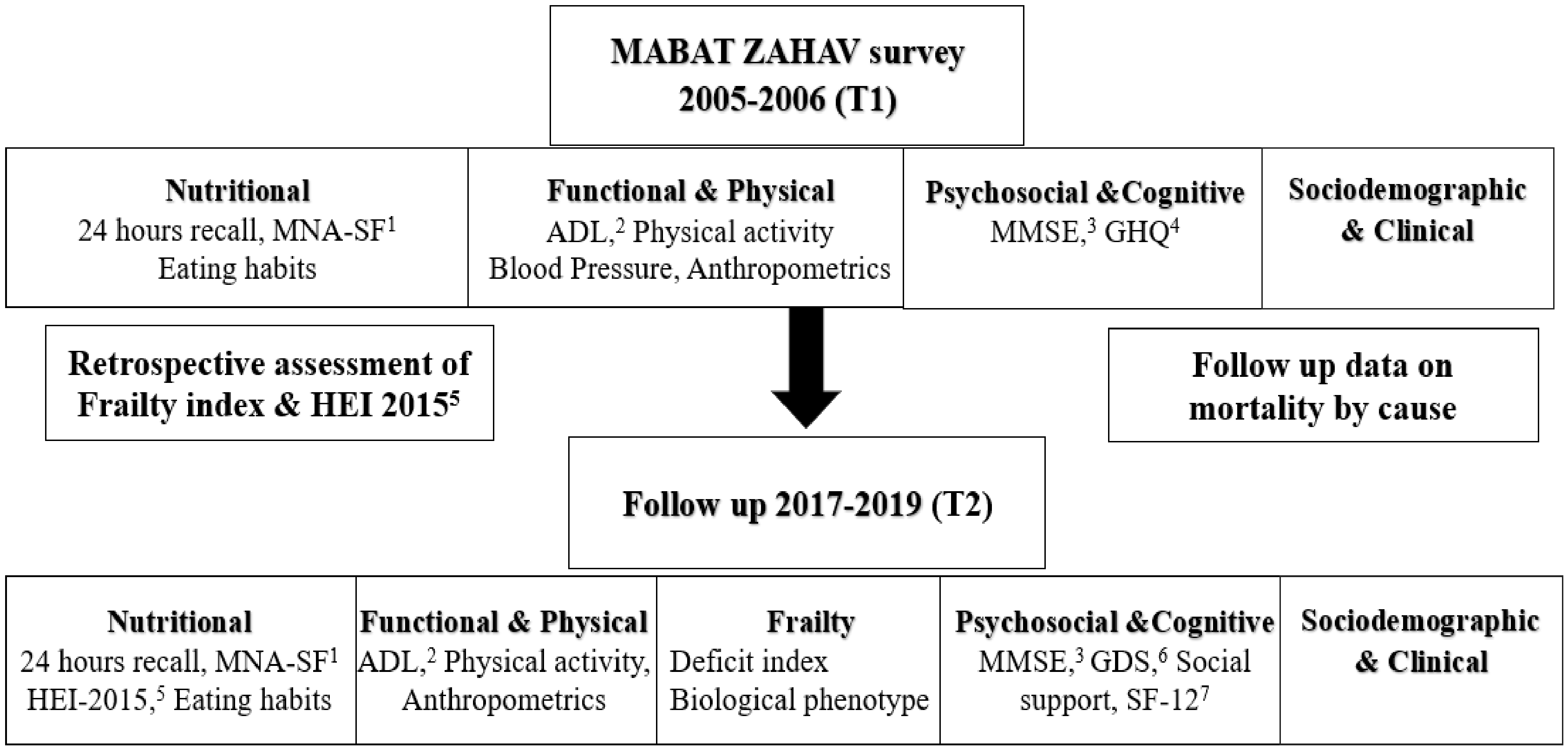

Notes: SA= successful aging; HEI= healthy eating index.[1]Mini Nutritional Assessment– Short Form; [2]Activities of Daily Living; [3]Mini Mental State Examination; [4] General Health Questionnaire; [5]Healthy Eating Index; [6]Geriatric Depression Scale; [7]Short Form of health-related-quality of life.

© 2022 Goshen A et al. *JAMA Network Open*.
